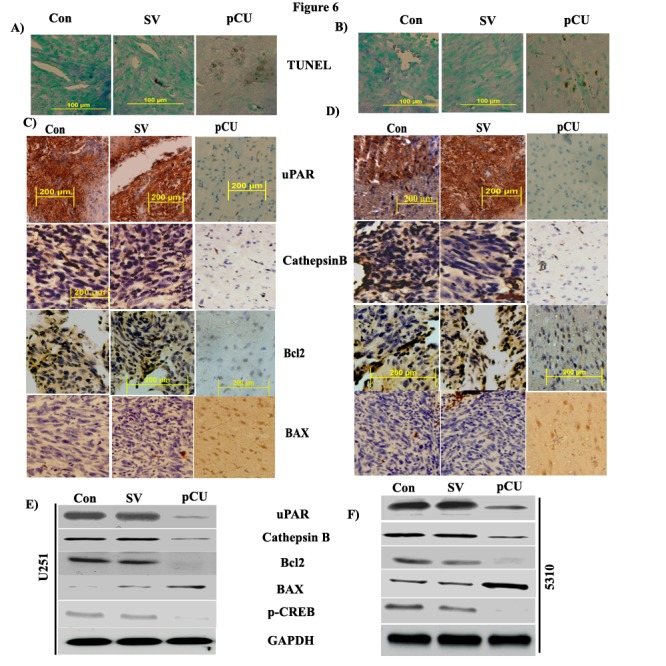# Correction: Downregulation of uPAR and Cathepsin B Induces Apoptosis via Regulation of Bcl-2 and Bax and Inhibition of the PI3K/Akt Pathway in Gliomas

**DOI:** 10.1371/annotation/bb2f92ec-2ac1-4952-b744-0519aeb10d1c

**Published:** 2014-01-02

**Authors:** Ramarao Malla, Sreelatha Gopinath, Kiranmai Alapati, Christopher S. Gondi, Meena Gujrati, Dzung H. Dinh, Sanjeeva Mohanam, Jasti S. Rao

Panels D and F of Figure 6 were duplicated. Please see the corrected Figure 6 here: 

**Figure:**